# The 7E Teaching Model in Emergency Obstetrics and Gynecology Training: Enhancing Clinical Competency in Residency Education

**DOI:** 10.1155/emmi/9349457

**Published:** 2026-01-30

**Authors:** Ling Yan, Tingting Liu, Linrui Wang, Jinke Li, Hefeng Zhang, Jiajin Zhu, Xiaoxue Wang, Kexin Tang, Dandan Zhang

**Affiliations:** ^1^ Department of Nursing, Shengjing Hospital of China Medical University, Shenyang, China, cmu.edu.cn; ^2^ Department of Obstetrics and Gynecology, Shengjing Hospital of China Medical University, Shenyang, China, cmu.edu.cn; ^3^ Department of Obstetrics and Gynecology, General Hospital of Fushun Mining Bureau of Liaoning Health Industry Group, Fushun, China; ^4^ Department of Health Management, Shengjing Hospital of China Medical University, Shenyang, China, cmu.edu.cn

**Keywords:** 7E teaching model, clinical competency, emergency obstetrics and gynecology, medical education, residency training

## Abstract

**Objective:**

To evaluate whether an 8‐week curriculum structured by the 7E teaching model is associated with improved emergency obstetrics and gynecology (OBGYN) competencies among first‐year residents.

**Methods:**

We conducted a prospective, single‐center, quasiexperimental consecutive‐cohort study at the Shengjing Hospital of China Medical University (September 2022–September 2024). Consecutive training‐year cohorts were compared: the 2022 cohort received traditional training (control, *n* = 32), and the 2023 cohort received the 7E‐based program (intervention, *n* = 31; elicit–engage–explore–explain–elaborate–evaluate–extend). Outcomes were mapped to Kirkpatrick’s four‐level model (reaction, learning, behavior, and results) and included satisfaction, written examination scores, OSCE performance, Mini‐CEX trajectories, and selected patient safety/process indicators.

**Results:**

Compared with the control group, residents in the 7E cohort reported higher satisfaction (35.10 ± 1.30 vs. 23.22 ± 1.84, *p* < 0.001; Cronbach’s *α* = 0.905), achieved higher written examination scores (71.23 ± 4.25 vs. 63.88 ± 5.71, *p* < 0.001), and demonstrated a higher OSCE excellence rate (80.65% vs. 15.63%, *p* < 0.001). Compliance with prespecified “golden‐hour” emergency process indicators was higher in the 7E cohort (87.09% vs. 59.38%, *p* = 0.025). Mini‐CEX trajectories showed a significant group × time interaction (*β* = −1.48 per week, 95% CI −1.75 to −1.21, *p* < 0.001), indicating faster skill improvement in the 7E cohort. Adherence to “golden‐hour” emergency process indicators was higher (87.09% vs. 59.38%, *p* = 0.025). Severe complications occurred in 1/31 (3.23%) versus 6/32 (18.75%), corresponding to a risk ratio = 0.17 (score‐based 95% CI 0.03–1.01; Fisher’s exact *p* = 0.104).

**Conclusion:**

The 7E‐structured curriculum was associated with improved satisfaction and performance across multiple competency domains. The 7E model shows promise as a structured paradigm for competency‐based emergency OBGYN education, although causal inferences are limited by the nonrandomized design and single‐institution context.

## 1. Introduction

Emergency obstetrics and gynecology (OBGYN) is a high‐stakes field requiring precise, time‐sensitive decision‐making, advanced technical skills, and humanistic care. In China, approximately 75% of maternal mortality occurs during emergency interventions, most commonly due to postpartum hemorrhage, preeclampsia, and placental abruption [1]. These conditions often progress insidiously yet deteriorate rapidly, demanding efficient care within narrow therapeutic windows. The challenges have been amplified by the three‐child policy, which has led to a sharp rise in advanced maternal age (37.8% in 2023 [2]) and a 2.3‐fold increase in pregnancy‐related comorbidities over the past decade.

Despite these demands, current residency training programs face three major limitations. First, the traditional apprenticeship model emphasizes procedural repetition rather than critical reasoning. Only 58.3% of trainees consistently apply the subjective–objective–assessment–plan (SOAP) framework, while 37.6% fail to formulate differential diagnoses [3]. Second, training is fragmented, often confined within specialty boundaries, which hinders integrated clinical competency. For instance, managing a ruptured ectopic pregnancy requires collaboration across emergency medicine, radiology, and anesthesiology. Yet, curricula often focus narrowly on discipline‐specific knowledge, neglecting interdisciplinary teamwork. Third, deficiencies persist in the development of humanistic skills. A national survey found that 83.4% of medicolegal disputes in emergency care stemmed from communication failures, yet fewer than 40% of residency programs incorporate formal patient–physician communication training [4].

Globally, competency‐based medical education (CBME) has reshaped training standards, with the Accreditation Council for Graduate Medical Education (ACGME) emphasizing communication and systems‐based practice in acute care [5]. The 7E model—an expansion of the 5E inquiry cycle—offers a promising framework, structured around seven phases: elicit, engage, explore, explain, elaborate, evaluate, and extend [6]. By embedding clinical dilemmas into the learning process, the model promotes the integration of knowledge, technical skills, and professional values.

Although prior studies have demonstrated the effectiveness of the 7E model in areas such as diabetic foot ulcer prevention [7], its application in emergency obstetrics remains limited. Most research to date has emphasized isolated instructional strategies, such as simulation, rather than comprehensive pedagogical models.

To address these gaps, this study proposes a three‐dimensional educational framework based on the 7E model, tailored specifically for emergency OBGYN. The framework emphasizes (1) knowledge acquisition through inquiry‐based exploration of pathophysiology; (2) skill mastery via modular drills, high‐fidelity simulation, and supervised clinical escalation; and (3) professionalism development through systematic reinforcement of patient safety culture. We conducted a prospective, single‐center, quasiexperimental consecutive‐cohort study to examine whether implementation of a 7E‐structured curriculum is associated with improved resident outcomes. The findings provide preliminary evidence to inform curriculum design for emergency obstetric education; transferability to other institutions and specialties will depend on local resources and training systems.

## 2. Materials and Methods

### 2.1. Study Design

**FIGURE 1:**
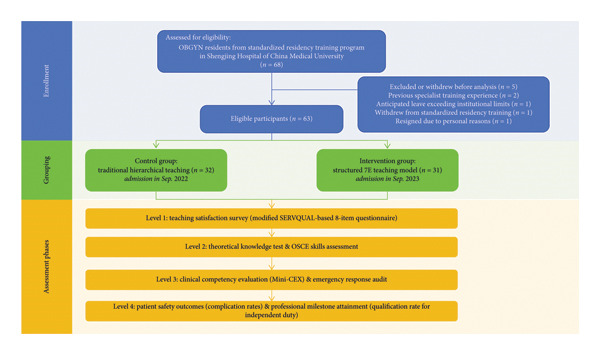
Participant flow diagram for the prospective, quasiexperimental consecutive‐cohort study.

This was a prospective, single‐center, quasiexperimental cohort study with a nonrandomized, consecutive‐cohort allocation (2022 control group; 2023 intervention group). The study was conducted at the Obstetrics and Gynecology (OBGYN) Residency Training Base of Shengjing Hospital, China Medical University, from September 2022 to September 2024 (Figure 1). The protocol was approved by the Institutional Ethics Committee (Approval No.: 2021PS744K), and written informed consent was obtained from all participants. Because allocation was determined by training year rather than randomization, we report the study as a quasiexperimental evaluation and avoid causal language beyond association.

### 2.2. Participants

First‐year OBGYN residents in the OBGYN program were enrolled through convenience sampling. The 2022 cohort served as the control group (*n* = 32), and the 2023 cohort served as the intervention group (*n* = 31). Inclusion criteria were completion of core rotations in emergency medicine and general surgery and voluntary participation. Exclusion criteria were (1) prior specialized training in emergency obstetrics; (2) absence from training for > 5 days; and (3) withdrawal from the residency program. Baseline characteristics, including age, gender, and educational background, were comparable between groups (*p* > 0.05). No formal baseline written pretest or baseline OSCE was administered before the 8‐week program; therefore, residual baseline differences (e.g., prior simulation exposure or emergency case exposure) cannot be fully excluded and are addressed as a limitation.

### 2.3. Intervention Measures

#### 2.3.1. Control Group

Residents in the control group received conventional tiered training, comprising 12 h of theoretical instruction and 18 h of skill‐based training over two months. Theoretical sessions followed the textbook *Clinical Obstetrics and Gynecology Emergency* (Chinese Clinical Emergency Series), with two weekly lectures covering key obstetric emergencies. Skill sessions were conducted at the Clinical Skills Center and included postpartum hemorrhage suturing and fetal heart rate monitoring interpretation, following a “demonstration–guided practice–independent operation” approach. For conditions such as placental abruption, teaching primarily emphasized guideline‐based recognition points, standard management steps, and key procedural demonstrations, without a fixed inquiry sequence or structured role‐based team rehearsal as an organizing framework. Residents participated in monthly emergency night shifts, managing approximately 8–10 cases per month under senior physician supervision.

#### 2.3.2. Intervention Group

The intervention group received an 8 week curriculum explicitly structured by the 7E sequence. The teaching team consisted of one chief physician, two attending physicians, one medical education specialist, and three nurses, all of whom completed standardized training in the 7E method (100% pass rate). The program included 8 h/week of structured sessions (64 h total) and was organized into three phases: Weeks 1 and 2 (elicit–engage–explore–explain), weeks 3–6 (elaborate–evaluate), and weeks 7 and 8 (extend with clinical transfer and reflection). Importantly, high‐fidelity simulation, skills checklists, and video debriefing were intentionally embedded within specific 7E phases (e.g., simulation and stress inoculation in elaborate; structured feedback and performance metrics in evaluate; workplace transfer tasks in extend) to operationalize a consistent learning progression from activation of prior knowledge to clinical transfer. Placental abruption was used as a representative scenario to illustrate the framework (Table 1).

**TABLE 1:** Implementation framework of the 7E teaching model using placental abruption as a representative scenario.

Teaching phase	Teaching method	Teaching content
Elicit	Clinical Scenario Creation	Playing a video of a patient with placental abruption presenting to the emergency department (including a medical‐patient conflict scenario)
	Cognitive Conflict Introduction	Displaying ultrasound images of the same case at different stages (from concealed to overt abruption)
	Triggering Question Chains	Constructing key question chains: “How to differentiate non‐specific abdominal pain?” “How to handle family refusal of surgery?”

Engage	Role Allocation	Each group of four participants plays the roles of attending physician, resident physician, midwife, and family member
	Task List	Completing focused physical examination (uterine tension, fetal heart rate changes) within 10 min; communicating with the “family” to obtain informed consent

Explore	Evidence‐Based Learning	Reviewing the grading criteria for placental abruption in UpToDate and FIGO guidelines
	Plan Development	Developing treatment flowcharts for different gestational weeks (28 weeks vs. 36 weeks)
	Overcoming Difficulties	Practicing DIC resuscitation using the Gaumard VICTORIA virtual simulation system

Explain	Group Presentation	Reporting cases using the SBAR model (Situation‐Background‐Assessment‐Recommendation)
	Expert Demonstration	Demonstrating multidisciplinary communication skills in emergency cesarean section decision‐making by an attending physician
	Cognitive Correction	Comparing and analyzing differences between trainee plans and actual resuscitation cases

Elaborate	High‐Fidelity Simulation	Practicing resuscitation of placental abruption with fetal distress on the SimMom® model
	Stress Testing	Introducing unexpected situations (e.g., blood bank emergency, anesthesia delay)
	Iterative Optimization	Reviewing team performance based on video playback

Evaluate	Formative Assessment	Assessing surgical operation standards using the OSATS scale
	Multidimensional Feedback	Patients′ Likert rating (1–5) of physician–patient communication
	Objective Indicators	Recording decision‐making time from triage to fetal delivery (minutes)

Extend	Community Linkage	Participating in home monitoring guidance for pregnant women with hypertension
	Policy Discussion	Analyzing the “Expert Consensus on the Construction of Obstetric Rapid Response Teams”
	Reflective Writing	Writing a reflective report on “Challenges in Clinical Decision‐Making”

### 2.4. Evaluation System

Training outcomes were assessed using Kirkpatrick’s four‐level evaluation model: Level 1 (Reaction), Level 2 (Learning), Level 3 (Behavior), and Level 4 (Results).

#### 2.4.1. Level 1: Reaction (Satisfaction)

Teaching satisfaction was measured using a modified SERVQUAL‐based questionnaire including eight dimensions (course design, faculty teaching ability, simulation fidelity, clinical decision‐making guidance, formative assessment, interprofessional collaboration, support for self‐directed learning, and balance of pressure/support). Each item was rated on a 5‐point Likert scale (1 = very dissatisfied; 5 = very satisfied). In the present study, the questionnaire demonstrated strong internal consistency (Cronbach’s *α* = 0.905).

#### 2.4.2. Level 2: Learning (Written Examination and OSCE)

Knowledge acquisition was assessed using an 80‐item multiple‐choice written examination (maximum score 80), aligned to program objectives and covering basic knowledge, differential diagnosis, treatment principles, and emergency indications. Practical competence was evaluated using an Objective Structured Clinical Examination (OSCE) consisting of five emergency stations (each scored on a 0–100 scale). The overall OSCE score was calculated as the average of the five station scores. OSCE excellence was defined as achieving ≥ 75 points in at least four stations. The written examination blueprint was mapped to program objectives; items were drafted by faculty and reviewed by two senior physicians for content relevance and clarity. OSCE stations were designed around guideline‐consistent critical actions and local emergency workflows, using standardized checklists and predefined scoring rubrics. Examiners participated in a calibration session prior to assessment. Because cohorts were separated by training year, complete blinding to cohort year was not feasible; examiners were not informed of the study hypothesis and applied the prespecified scoring rubrics uniformly.

#### 2.4.3. Level 3: Behavior (Mini‐CEX Trajectory and Process Adherence)

Workplace‐oriented performance was assessed using weekly Mini‐CEX evaluations during the 8 week program. Each assessment included seven domains scored on a 9‐point scale (medical interviewing, physical examination, clinical judgment, clinical management, communication, professionalism, and organization; total score range: 7–63), summarized as a total Mini‐CEX score. Compliance with “golden‐hour” emergency process indicators was assessed during real‐case management. The Mini‐CEX assessments were completed by attending physicians acting as clinical supervisors. All assessors received a standardized orientation/calibration session on the Mini‐CEX rubric and rating anchors. The seven‐domain Mini‐CEX scale demonstrated strong internal consistency across observations (Cronbach’s *α* = 0.907). Because the cohorts were separated by training year, assessors could not be blinded to cohort year; residents typically received ratings from more than one assessor over the 8 weeks due to scheduling, although the exact number of distinct raters per resident was not systematically recorded.

#### 2.4.4. Level 4: Results (Patient Safety and Professional Development)

Patient safety outcomes were assessed by comparing the incidence of severe complications between groups, and professional development was assessed using the independent on‐call qualification rate at six months after training.

### 2.5. Statistical Analysis

Data were analyzed using SPSS 29.0 and R. Continuous variables are reported as mean ± standard deviation (SD) and compared using independent‐samples *t* tests (two‐sided). Categorical variables are reported as *n* (%) and compared using *χ* ^2^ tests or Fisher’s exact tests, as appropriate. For Mini‐CEX repeated measures, generalized estimating equations (GEEs) with Gaussian distribution and an exchangeable working correlation structure were used to model Mini‐CEX total scores over time, with predictors including group, time (weekly assessment occasion, weeks 1–8), and a group × time interaction. For severe complications (rare events), risk ratios (RRs) were calculated from raw event counts; 95% confidence intervals were computed using the score‐based method for the ratio of two independent proportions, and *p* values were obtained using Fisher’s exact test. Statistical significance was defined as *p* < 0.05.

## 3. Results

### 3.1. Study Participants and Flow

A total of 68 residents were initially enrolled. Three were excluded (two with prior specialized training and one with anticipated prolonged absence), and two withdrew during the study (one resigned from residency, one left for family reasons). Thus, 63 participants completed the trial (31 in the intervention group and 32 in the control group) (Figure 1). Baseline demographic characteristics, including age, gender, and educational background, did not differ significantly between groups (*p* > 0.05) (Table 2).

**TABLE 2:** Baseline demographic characteristics of resident physicians in the intervention and control groups.

Variable	Intervention group (*n* = 31)	Control group (*n* = 32)	Statistic (*t*/*χ* ^2^)	*p*
Age (years)	24.48 ± 2.204	24.97 ± 1.84	0.949	0.346
Gender			0.502	0.478
Male	3 (9.7%)	5 (15.6%)		
Female	28 (90.3%)	27 (84.4%)		
Educational Background			0.337	0.561
Master’s Degree	5 (16.1%)	7 (21.9%)		
Bachelor’s Degree	26 (83.9%)	25 (78.1%)		

### 3.2. Outcomes Based on Kirkpatrick’s Four‐Level Evaluation

#### 3.2.1. Level 1: Reaction

Teaching satisfaction was higher in the intervention group compared with that in the control group (35.10 ± 1.30 vs. 23.22 ± 1.84; *p* < 0.001). The largest differences were observed in the dimensions of scientific nature of course design (4.74 ± 0.44 vs. 3.19 ± 0.64, *p* < 0.001) and effectiveness of clinical decision‐making guidance (4.71 ± 0.53 vs. 2.88 ± 0.66, *p* < 0.001) (Table 3). The satisfaction questionnaire demonstrated strong internal consistency (Cronbach’s *α* = 0.905).

**TABLE 3:** Comparison of teaching satisfaction between groups using the modified SERVQUAL‐based questionnaire.

Dimension	Intervention group (*n* = 31)	Control group (*n* = 32)	*t*	*p*
Scientific Nature of Course Design	4.74 ± 0.44	3.19 ± 0.64	11.11	< 0.001
Teaching Ability of Faculty	4.84 ± 0.37	3.22 ± 0.79	10.32	< 0.001
Fidelity of Simulation Equipment	4.35 ± 0.66	2.62 ± 0.83	9.11	< 0.001
Effectiveness of Clinical Decision‐Making Guidance	4.71 ± 0.53	2.88 ± 0.66	12.15	< 0.001
Formative Assessment Mechanism	4.26 ± 0.63	2.75 ± 0.67	9.18	< 0.001
Interprofessional Collaboration Training	4.23 ± 0.43	3.06 ± 0.56	9.22	< 0.001
Support for Self‐Directed Learning	3.90 ± 0.65	2.88 ± 0.91	5.16	< 0.001
Balance of Pressure and Support	4.06 ± 0.44	2.62 ± 0.91	7.97	< 0.001
Total Score	35.10 ± 1.30	23.22 ± 1.84	29.46	< 0.001

*Note:* Values are mean ± SD. Cronbach’s *α* for the 8‐item satisfaction instrument = 0.905.

#### 3.2.2. Level 2: Learning

Residents in the intervention group achieved higher written examination scores than those in the control group (71.23 ± 4.25 vs. 63.88 ± 5.71, *t* = 5.79, *p* < 0.001). Among subdomains, the between‐group differences were most prominent for differential diagnosis (17.65 ± 1.68 vs. 14.91 ± 2.70, *p* < 0.001) and emergency indication judgment (17.94 ± 1.75 vs. 16.19 ± 2.05, *p* < 0.001) (Table 4).

**TABLE 4:** Comparison of theoretical and skill assessment scores between groups.

Domain/station	Intervention group (*n* = 31)	Control group (*n* = 32)	Statistic (*t*/ *χ* ^2^)	*p*
Written examination (max 80 points)				
Basic Knowledge	17.55 ± 2.05	16.13 ± 2.86	2.27	0.027
Differential Diagnosis	17.65 ± 1.68	14.91 ± 2.70	4.81	< 0.001
Treatment Principles	18.10 ± 1.99	16.66 ± 2.74	2.38	0.020
Emergency Indications	17.94 ± 1.75	16.19 ± 2.05	3.63	< 0.001
Total Score	71.23 ± 4.25	63.88 ± 5.71	5.78	< 0.001

OSCE station scores (each station 0–100; overall score = mean of 5 stations)				
Hemorrhagic Shock Resuscitation	83.29 ± 7.10	80.06 ± 4.61	2.15	0.036
Eclampsia Resuscitation	82.06 ± 4.93	77.41 ± 7.00	3.05	0.003
Amniotic Fluid Embolism Resuscitation	81.90 ± 8.18	69.78 ± 6.53	6.51	< 0.001
Postpartum Hemorrhage Resuscitation	87.16 ± 6.93	76.53 ± 9.29	5.14	< 0.001
Neonatal Asphyxia Resuscitation	79.65 ± 7.64	76.25 ± 5.84	1.99	0.052
Total OSCE score (Sum of 5 stations × 0.2)	82.81 ± 3.59	76.01 ± 3.08	8.08	< 0.001
OSCE excellence rate	25/31 (80.65%)	5/32 (15.63%)	26.69	< 0.001

*Note:* Values are mean ± SD. OSCE excellence defined as ≥ 75 points in at least four stations.

In the OSCE, the intervention group achieved higher performance across stations, with particularly large differences in amniotic fluid embolism resuscitation (81.90 ± 8.18 vs. 69.78 ± 6.53, *p* < 0.001) and postpartum hemorrhage resuscitation (87.16 ± 6.93 vs. 76.53 ± 9.29, *p* < 0.001). Improvements were smaller for stations that are typically supported by more established, highly standardized training traditions (e.g., hemorrhagic shock and eclampsia). Notably, neonatal asphyxia resuscitation showed a numerically higher mean score in the intervention group, but the difference did not reach statistical significance (79.65 ± 7.64 vs. 76.25 ± 5.84, *p* = 0.053). The overall OSCE score was higher in the intervention group (82.81 ± 3.59 vs. 76.01 ± 3.08, *p* < 0.001). The OSCE excellence rate (≥ 75 points in at least four stations) was 80.65% (25/31) in the intervention group versus 15.63% (5/32) in the control group (*p* < 0.001) (Table 4).

#### 3.2.3. Level 3: Behavior

**FIGURE 2:**
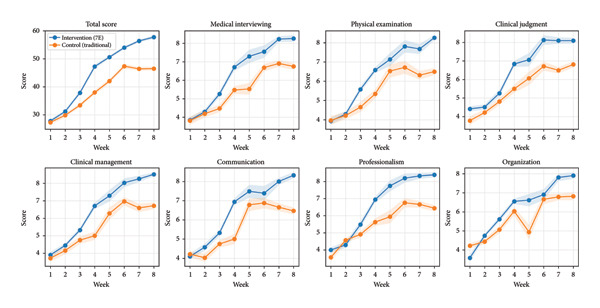
Learning curves of weekly Mini‐CEX total scores over the 8‐week program. The *x*‐axis indicates the weekly Mini‐CEX assessment occasion (week 1–week 8). Lines represent the intervention group (red, *n* = 31) and the control group (blue, *n* = 32).

Mini‐CEX scores were assessed weekly over the 8‐week program. In the GEE model, there was a significant time effect and a significant group × time interaction, indicating different performance trajectories between cohorts. Using “time” as the weekly Mini‐CEX assessment occasion (weeks 1–8), the interaction term was statistically significant (*β* = −1.479, SE = 0.137, *p* < 0.001), consistent with faster improvement in the intervention group and a comparatively attenuated slope in the control group over time (Table 5; Figure 2).

**TABLE 5:** Time‐effect analysis of Mini‐CEX evaluations using generalized estimating equations.

Parameter	*β*	Standard error	95% CI	*p*
Intercept	24.575	0.502	23.592 to 25.558	< 0.001
Group (2 vs. 1)	0.161	0.744	−1.298 to 1.620	0.829
times	4.616	0.093	4.433 to 4.798	< 0.001
Group ∗ times (2 vs. 1)	−1.479	0.137	−1.748 to −1.210	< 0.001

*Note:* Outcome: Mini‐CEX total score (range 7–63). Time = weekly assessment occasion (week 1–week 8). Reference group: groups = 1 (intervention group); groups = 2 (control group). Line Intercept\times\Group^∗^times.

In real emergency case management, compliance with prespecified golden‐hour emergency process indicators was higher in the intervention group (87.09%, 27/31) than in the control group (59.38%, 19/32) (*χ* ^2^ = 5.03, *p* = 0.025).

#### 3.2.4. Level 4: Results

Patient safety outcomes favored the intervention group, but event counts were low. Severe complications occurred in 1/31 (3.23%) in the intervention group and 6/32 (18.75%) in the control group, corresponding to an RR of 0.17 with a 95% confidence interval of 0.03–1.01. Given the low number of events, this estimate is imprecise and did not reach statistical significance on Fisher’s exact test (*p* = 0.104). Therefore, the finding is interpreted as an association requiring confirmation in larger, contemporaneous studies.

Six months after training, the independent on‐call qualification rate was higher in the intervention group (90.32%, 28/31) than in the control group (71.88%, 23/32), although the difference did not reach statistical significance (*χ* ^2^ = 2.38, *p* = 0.122).

### 3.3. Adverse Events and Protocol Deviations

One stress‐related response (tachycardia > 120 bpm) was reported in both groups during simulation training; symptoms resolved after pausing the session. The control group also experienced two procedural errors, neither of which caused patient harm. No protocol deviations affected final data analysis.

## 4. Discussion

### 4.1. Advantages of the 7E Teaching Model in Emergency OBGYN

In this prospective, single‐center, quasiexperimental comparison of consecutive resident cohorts, an 8‐week curriculum structured by the 7E model was associated with higher learner satisfaction and improved performance on written examinations, OSCE stations, and longitudinal Mini‐CEX trajectories. These findings support the potential value of a theory‐driven instructional architecture for competency‐based emergency OBGYN education.

A key contribution of this work is to clarify that the observed gains are unlikely to be attributable to a single educational technique (e.g., simulation, checklist‐based assessment, or video debriefing), because many of these components are increasingly standard in emergency obstetric training. Rather, the distinguishing feature of the intervention is the explicit 7E sequence as the organizing logic of the curriculum. The 7E structure operationalizes a progression from activation of prior knowledge and cognitive conflict (elicit/engage), to guided inquiry and conceptual modeling (explore/explain), to progressively challenging practice and stress inoculation (elaborate), followed by structured feedback anchored to performance criteria (evaluate), and finally to transfer tasks and reflection in workplace contexts (extend) [8, 9]. We hypothesize that this consistent sequence strengthens integration across cognitive, psychomotor, and professional domains more effectively than deploying similar educational tools without an explicit inquiry‐to‐transfer pathway.

This perspective also helps position the study relative to prior emergency obstetric education reports: many interventions demonstrate that simulation and debriefing improve specific procedural or crisis‐management skills, but fewer studies examine a curriculum‐level architecture spanning multiple Kirkpatrick levels, including repeated workplace assessment (Mini‐CEX trajectories) and selected process/patient safety indicators. The present findings suggest that applying the 7E structure as an overarching curriculum design may offer incremental value beyond technique‐level interventions.

### 4.2. Promotion of Clinical Reasoning and Decision‐Making

Emergency OBGYN care requires rapid hypothesis generation, prioritization under uncertainty, and time‐sensitive escalation [10]. In the present curriculum, the 7E sequence was designed to repeatedly connect (i) problem representation and cognitive conflict (elicit/engage), (ii) evidence‐based exploration and explicit reasoning articulation (explore/explain), and (iii) deliberate practice with escalating complexity and interruptions (elaborate), followed by structured evaluation against observable standards (evaluate) [11]. This mechanism plausibly aligns with the larger between‐group differences observed in written exam subdomains that depend heavily on reasoning—particularly differential diagnosis and emergency indication judgment—rather than rote recall [12].

The OSCE station pattern further supports this interpretation. The largest between‐group differences were observed in amniotic fluid embolism and postpartum hemorrhage—scenarios characterized by low tolerance for diagnostic delay, rapidly evolving physiology, and high demands for coordinated decision‐making. In contrast, the smaller incremental gains for hemorrhagic shock and eclampsia may reflect relatively stronger baseline preparedness owing to more established training traditions and guideline standardization, potentially creating a ceiling effect that constrains additional improvement within a short time frame.

Importantly, neonatal asphyxia resuscitation demonstrated a numerically higher mean score in the intervention group but did not reach statistical significance in our dataset (two‐sided *p* = 0.052). This borderline result may reflect the same ceiling effect phenomenon, limited statistical power for modest station‐level effects, or variability in prior exposure to neonatal resuscitation training.

### 4.3. Cultivation of Professionalism and Humanistic Competency

Emergency settings also test professionalism, communication, and team‐based safety behaviors. The intervention deliberately embedded communication and interprofessional role clarity within the 7E structure, including explicit role allocation and task lists during engage, collaborative planning in explore, and structured feedback during evaluate [13]. The extend phase emphasized clinical transfer and reflective practice, which may support sustained attention to safety‐oriented behaviors (e.g., escalation, checklist adherence, and structured communication under time pressure) [14].

Consistent with this intent, longitudinal Mini‐CEX analysis demonstrated significantly steeper improvement trajectories for the 7E cohort across the 8 week program, suggesting that structured sequencing combined with repeated observation and feedback may be associated with improved workplace‐oriented performance development [15].

### 4.4. Limitations and Future Directions

Several limitations require careful interpretation. First, allocation by training year (2022 control vs. 2023 intervention) introduces potential cohort and temporal confounding. Although baseline demographics were similar, we did not administer a baseline knowledge or skills pretest; therefore, residual baseline differences (e.g., prior exposure to simulation, emergency case mix, or informal learning opportunities) cannot be ruled out. Additionally, institutional changes over time (workflow refinement, faculty experience, evolving guidelines) may have contributed to differences between cohorts. For these reasons, the results should be interpreted as associations, and causal inference should be avoided. As the same, assessor‐related bias is possible. Because cohorts were separated by year, complete blinding of examiners and clinical evaluators may not have been feasible, and knowledge of cohort status could influence ratings, particularly for workplace‐based assessments such as Mini‐CEX.

Then, the patient safety findings were based on low event counts. Although severe complications were numerically lower in the 7E cohort (1/31 vs. 6/32), the resulting effect estimate was imprecise with a wide confidence interval, emphasizing the need for larger samples and contemporaneous designs to confirm clinical outcome effects. Furthermore, feasibility and scalability deserve explicit attention. The 7E program required substantially more structured teaching time (64 h vs. 30 h) and an interprofessional teaching team, along with simulation resources and debriefing time. These requirements (faculty protected time, simulator access, scheduling flexibility, assessor training) may limit adoption in lower‐resource settings. Implementation studies should explore streamlined versions of the 7E sequence, quantify resource utilization, and evaluate cost‐effectiveness.

Finally, this was a single‐center study within a specific national and institutional context. Generalizability to other health systems, specialties, and residency structures is limited. Future research should include multicenter studies, contemporaneous controls, and—where feasible—randomized designs, with longer follow‐up to assess durability of learning and downstream patient outcomes.

## 5. Conclusion

In this single‐center, prospective, quasiexperimental comparison of consecutive first‐year OBGYN resident cohorts, an 8 week curriculum structured by the 7E teaching model was associated with higher learner satisfaction, higher written examination scores, improved OSCE performance, and a steeper Mini‐CEX improvement trajectory over time. The intervention group also showed higher adherence to prespecified “golden‐hour” emergency process indicators, while differences in rare patient safety events were directionally favorable but imprecise due to low event counts.

Overall, the 7E model shows promise as a structured instructional paradigm for competency‐based emergency OBGYN education, particularly for integrating clinical reasoning, team‐based crisis management, and transfer to workplace practice. Given the nonrandomized, noncontemporaneous cohort design, single‐center context, and resource requirements, future multicenter contemporaneous studies—ideally with stronger causal designs where feasible—are needed to confirm effectiveness, clarify mechanisms, and evaluate feasibility and longer term clinical outcomes.

**Nomenclature**
CBME: Competency‐based medical education
ACGME: Accreditation Council for Graduate Medical Education
OSCE: Objective structured clinical examination
Mini‐CEX: Mini‐clinical evaluation exercise
GEEs: Generalized estimating equations
RR: Relative risk
CI: Confidence interval
SERVQUAL: Service quality model

## Author Contributions

Ling Yan, Tingting Liu, and Dandan Zhang designed the study and drafted the manuscript. Tingting Liu, Linrui Wang, Xiaoxue Wang, and Dandan Zhang undertook the teaching content. Jinke Li, Hefeng Zhang, Kexin Tang, and Jiajin Zhu designed the statistical analysis plan. Dandan Zhang has reviewed the manuscript.

## Funding

This study was supported in part by the Medical Education Research Project of Liaoning Province (Nos. 2022‐005‐003 and 2024‐004‐03), the Liaoning Graduate Education Teaching Reform Research Project (No. LNYJG2024214), and “14th Five‐Year Plan” institutional‐level teaching reform project of the Second Clinical College of China Medical University (SJJG‐2024QN11).

## Ethics Statement

This study was approved by the Ethics Committee of the Shengjing Hospital of China Medical University (No. 2021PS746K, Date: 07/10/2021). Written informed consent was obtained from all participants.

## Consent

Please see Ethics Statement.

## Conflicts of Interest

The authors declare no conflicts of interest.

## Data Availability

The datasets generated and analyzed during the current study are available from the corresponding author upon reasonable request.
